# Human-guided synthesis planning *via* prompting†

**DOI:** 10.1039/d5sc00927h

**Published:** 2025-07-14

**Authors:** Annie M. Westerlund, Lakshidaa Saigiridharan, Samuel Genheden

**Affiliations:** a Molecular AI, Discovery Sciences, R&D, AstraZeneca 43183 Gothenburg Sweden

## Abstract

Contemporary multistep retrosynthesis tools such as AiZynthFinder, which are frequently used by chemists, generate solved routes for the majority of target molecules, but do not consider the prior knowledge of the chemist, including specific bonds that should disconnect or remain connected throughout the routes. Such knowledge is for example integral when planning a joint synthesis route for a set of similar molecules where common disconnection sites can be identified across the molecules. Here, we present a novel strategy in AiZynthFinder for human-guided multistep retrosynthesis *via* prompting. This includes a filter for discarding reactions that violate bonds to freeze constraints. Furthermore, we benchmark four possible strategies for breaking selected bonds in the search for synthetic routes, and show that a combination of a disconnection-aware transformer and a multi-objective search generates routes which satisfy bond constraints for more targets in the PaRoutes dataset compared to the standard search (75.57% *vs.* 54.80%). Finally, we apply the strategy on a set of drug molecules to exemplify a real-world scenario. Our novel approach enables building a short joint synthesis route that satisfies the given bond constraints and covers eight of the ten molecules, demonstrating the added value of incorporating human prior knowledge in synthesis planning.

## Abstract 2

This work presents a novel strategy for human-guided multistep retrosynthesis *via* prompting, using a disconnection-aware transformer and multi-objective Monte Carlo Tree Search.

## Graphical abstract

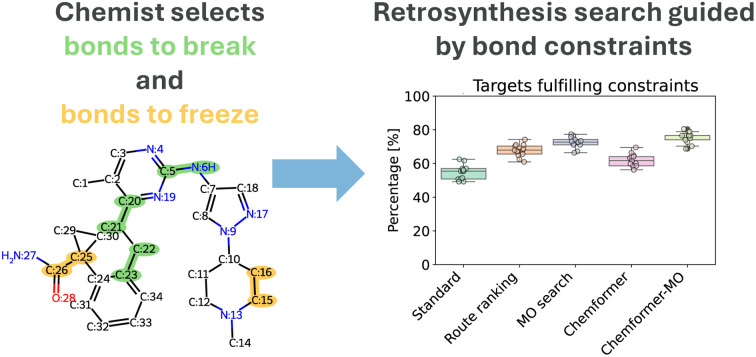

## Introduction

The synthesis of chemical matter is paramount in pharmaceutics, agriculture, and food industries. Retrosynthesis^1,2^ is a systematic approach commonly used for this purpose. It works by identifying potential disconnection sites in a target molecule (the product) to predict possible building block molecules (reactants) that could be used to make the product. The target molecule and subsequent intermediates are broken down iteratively until arriving at commercial starting materials. AI-driven retrosynthesis prediction,^3,4^ based on deep neural networks and search algorithms such as Monte Carlo Tree Search (MCTS), has become an intense area of research in the last decade. Several models and tools have been developed to speed up the synthesis-planning process.^3,5–16^ AiZynthFinder,^5,17^ for instance, is frequently used by chemists in industrial projects.^13,18^ Although AI-assisted retrosynthesis shows promise to become standard practice, a few challenges still remain, which should be solved before reaching its full potential and the generated synthesis plans can be used directly in the lab.

One of the challenges commonly occurring in drug discovery is the prediction of synthesis routes for a set of target molecules with shared intermediates. However, off-the-shelf AI-driven synthesis planning tools, such as AiZynthFinder, are typically unaware of concepts such as “multiple targets” or “shared intermediates”. The challenge has previously been approached either through a recursive search on a complete reaction network to generate cost-effective and diverse synthesis routes,^19,20^ or as a postprocessing step of retrosynthesis searches by carrying out multi-objective optimization on the generated reaction networks.^21,22^ The latter is naturally dependent on the quality of the set of routes generated by retrosynthesis. Moreover, multistep retrosynthesis which considers the prior knowledge of the chemist who carries out the experiments in the lab is an important topic. The chemist could, for example, be interested in breaking a specific bond, or in keeping certain bonds or moieties frozen in the generated synthesis route. This would be especially beneficial when planning a joint synthesis route for a set of similar compounds where common disconnection sites can be identified across the compounds.

Here, we present a novel strategy in AiZynthFinder for human-guided multistep retrosynthesis *via* prompting. The chemists provide input on what bonds should be disconnected (*bonds to break*) or remain connected (*bonds to freeze*) in the synthesis route as prompts to the tool. In its standard configuration, AiZynthFinder uses a so-called template-based model to suggest chemical disconnections and MCTS guided by a simple objective to break down the target molecule. To keep bonds frozen, any single step predictions violating the *bonds to freeze* constraints are filtered out. This functionality is referred to as the frozen bonds filter. For breaking user-specified bonds, we investigate two possible approaches to disconnection-aware multistep retrosynthesis *via* prompting. First, a novel score that favors routes satisfying the *bonds to break* constraints early in the search tree (see Methods; Broken bonds score). We integrate this score in the tree search using a recently developed multi-objective (MO) MCTS algorithm.^23,24^ Second, we introduce a framework where a disconnection-aware transformer, which is a prompt-based language model for disconnecting tagged *bonds to break*,^25,26^ is combined with the template-based model to allow for human-guided multistep retrosynthesis. The reader is referred to Methods; *Disconnection-aware Chemformer in multistep retrosynthesis* for details on how the two models are integrated in the multistep retrosynthesis framework.

We benchmark the novel approach on the PaRoutes set-n1 (ref. 27) and Reaxys-JMC (*Journal of Medicinal Chemistry*)^28^ datasets, which consist of known synthesis routes either from patents or the literature. Finally, we showcase a project-specific application with ten similar target compounds. Overall, the work presented here provides a novel approach for human-driven multistep retrosynthesis *via* prompting.

## Related studies

Our work focuses on prompting to guide multistep retrosynthesis searches. In this section, we contextualize the advances presented here and contrast our work to the work reported in the literature.

First, recent approaches have been reported for finding common intermediates and joint synthesis routes. One approach leveraged synthesis routes from AI-driven synthesis planning tools and carried out multi-objective optimization on the generated reaction networks.^21,22^ A recent alternative approach constructed a search tree for multiple targets at once using only the single step model scores.^29^ These tools are promising for automatically constructing a joint synthesis route for multiple targets. Although such methods do not allow the user to make suggestions of bonds to break or freeze on their own, they could in principle be used in conjunction with our human-guided retrosynthesis search in a workflow for creating joint synthesis routes for multiple targets given bond constraints.

Second, advances have been made for the purpose of prompting single step predictions to disconnect *bonds to break*. Recently, a disconnection-aware transformer model was proposed to guide single step predictions by tagging the disconnection site in the SMILES string prior to feeding it to the model.^25,26^ The first introduction of a disconnection-aware transformer demonstrated the ability of the model to learn to recognize the tags and disconnect the corresponding bonds.^26^ It was extensively benchmarked on single step predictions compared to a baseline retrosynthesis transformer in order to manifest its robustness and accuracy. In addition, the authors introduced a model for automatic tagging of reaction centers. The automatic tagging aims to step away from human-guided retrosynthesis and aids the disconnection-aware model to automatically generate more diverse predictions.^26^ In contrast to our work, the first disconnection-aware transformer was only used and evaluated for the purpose of single step retrosynthesis. Later, an alternative disconnection-aware transformer was used in a multistep search by pairing it with automatic tagging in each single-step iteration.^25^ Unlike the work presented here, automatic tagging of reaction centers was used to boost diversity. Hence, human-directed prompting of *bonds to break* in a multistep framework was not explored.

In contrast to earlier work on disconnection-aware transformers which only considered isolated single step predictions, we have developed two approaches for human-guided multistep retrosynthesis searches *via* prompting. We thus focus on generating synthesis routes, rather than single reaction predictions. The first approach is the novel broken bonds score which is used in a multi-objective search. The score enables guiding synthesis planning with prompted bond constraints without altering the single step model. Our second approach relies on the previously proposed disconnection-aware transformer. To this end, we introduce novel extensions to the single step model. For instance, several steps may be required to break the bonds, and hence the disconnection site tagging should be reliably propagated to the next steps in the synthesis route. We therefore constructed a custom expansion strategy (Table 1) which combines the predictions of the disconnection-aware model with predictions of the template-based model in order to allow further disconnections to reach solved routes. In addition to our two approaches, we introduce the frozen bonds filter. To our knowledge, approaches similar to the frozen bonds filter or the broken bonds score have not been reported prior to this work.

**Table 1:** Key terms and abbreviations used in this work

MCTS	Monte-Carlo Tree Search. Search algorithm used for multistep retrosynthesis. Iteratively predicting reactants given the current product molecule. A (single) objective, or score, is used to guide the search
MO-MCTS	Multi-objective Monte-Carlo Tree Search. MCTS with multiple objectives, or scores, to guide the search
Single step model	In each step of an AI-driven retrosynthesis search, predictions are carried out with a single step (machine learning) model which predicts reactants for a given product, either implicitly *via* a reaction template or explicitly *via* SMILES or molecule graphs
Expansion strategy	Used within the multistep retrosynthesis search to generate predictions with a single step model. It outputs model predictions which are ranked and, possibly, processed
AiZynthFinder	Software tool which uses MCTS, or MO-MCTS in combination with a single step model to generate multistep retrosynthesis predictions
Template-based model	The single-step model used in the standard AiZynthFinder search. Takes a molecule fingerprint as input and predicts reaction templates which can be applied to the product to obtain reactants
Chemformer	Transformer model which can be used as single step model. Retrosynthesis transformer models^12,25,26,30,31^ are trained on SMILES strings (reactant SMILES are predicted directly based on the product SMILES as input). This allows for non-chemical modifications to the input, including tagging atoms in bonds to break
Pareto front	An optimal balance of multiple objectives or scores, such that none of the scores can be improved without weakening the other score(s). The Pareto front is the set of solutions that fulfill this trade-off
Pareto rank	A ranking of Pareto fronts which are obtained by iteratively removing the primary Pareto front from the data. Ex: the set of samples in the new Pareto front obtained after removing the data on the first Pareto front corresponds to the second Pareto rank

Table 1 provides a glossary with key concepts and abbreviations used throughout this manuscript.

## Results and discussion

### Strategies for disconnection-aware multistep retrosynthesis

**Fig. 1:**
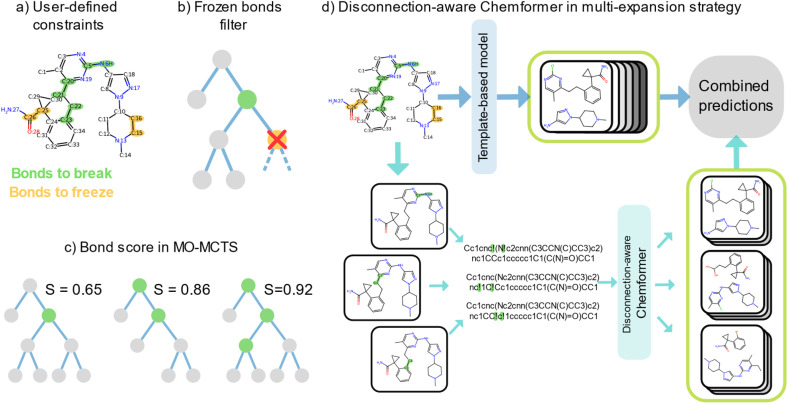
Depiction of the developed approaches. (a) Constraints are defined as “bonds to break” and “bonds to freeze”. (b) The frozen bonds filter directly removes a reaction which violates a “bonds to freeze” constraint. The nodes in the tree represent reactions. A green node represents a reaction that fulfills a “bonds to break” constraint while a yellow node represents a reaction that violates a “bonds to freeze” constraint. (c) The broken bonds score used in MO search rewards routes which disconnect many “bonds to break” early in the route. The green nodes represent reactions that fulfill “bonds to break” constraints. More satisfied “bonds to break” constraints earlier in the tree give a higher broken bonds score. (d) The disconnection-aware Chemformer is used in a multi-expansion strategy with the template-based model in AiZynthFinder. Each “bond to break” is tagged and handled separately by Chemformer. The predictions from both models are joined as a last step.

Fig. 1 illustrates the approaches for constrained synthesis planning. Prior to the retrosynthesis search, a user can define bond constraints as either bonds to break or bonds to freeze in the route (Fig. 1a). We treated the *bonds to freeze* constraints as hard constraints as it is simple to enforce which bonds should remain intact in each single step prediction. Conversely, we treated the *bonds to break* constraints as soft constraints in the search, since excluding predictions that violate such constraints would hinder the breaking of other bonds either before or after the specified *bonds to break*. Moreover, routes that only disconnect a subset of the *bonds to break* could still be useful as inspiration to the chemist. The *bonds to freeze* constraints were satisfied by a filter that directly removed any prediction which violated the constraints (Fig. 1b).

For the bonds to break constraints, we investigated two main approaches: (1) a novel broken bonds score to bias the tree search and route building steps and (2) a disconnection-aware Chemformer for boosting single step predictions. The reader is referred to the Methods section for technical details on the broken bonds score and disconnection-aware Chemformer.

#### Disconnection-aware search by scoring the routes

In the first approach, we devised a broken bonds score (Methods; Broken bonds score: eqn (1)) which returned a value between zero and one (Fig. 1c). Zero represents the case when none of the bonds to break were disconnected in any of the reactions, and one represents all bonds breaking in the first reaction. Specifically, given a set of bonds to break, a higher score is given to routes that disconnect those bonds early in the reaction tree. An example is shown in Fig. 1c, where the three *bonds to break* (defined in Fig. 1a) are disconnected in the third reaction tree, yielding a score of 0.92. In the first and second reaction trees in Fig. 1c, only a subset of the *bonds to break* was disconnected, leading to lower scores (0.65 and 0.86). A detailed explanation of the broken bonds score together with explicit examples of how it is computed is given in Methods; Broken bonds score. We used this score together with the default MCTS reward in AiZynthFinder (referred to as the state score),^32^ which favors short routes with a large fraction of precursors in stock.

Standard AiZynthFinder uses MCTS (Table 1) to combine single-step predictions. In standard MCTS, a single search objective is used (the state score). However, for retrosynthesis, there can be multiple objectives that describe route quality. For example, when prompting *bonds to break* to a retrosynthesis search, one objective is to favor routes which disconnect the prompted bonds (broken bonds score) and another objective is to favor routes which are solved and short (state score). Therefore, both the state score and broken bonds score are necessary to rank the routes in disconnection-aware retrosynthesis. In contrast to standard MCTS, multi-objective MCTS (MO-MCTS, Table 1) enables taking multiple objectives, or scores, into account when carrying out the retrosynthesis search. It has previously been explored for robotics environmental monitoring^24^ and was recently implemented for multistep retrosynthesis in AiZynthFinder.^23^ The extracted routes are part of the Pareto front (Table 1).

The reader is referred to the Methods section as well as the original publication of MO-MCTS in AiZynthFinder^23^ for technical details on how the MO-MCTS algorithm is implemented.

#### Disconnection-aware search by altering single-step predictions

In the second approach, a disconnection-aware Chemformer was used in a novel multi-expansion framework. Because disconnection-aware transformers^12,25^ alter single step predictions, we first evaluated the disconnection-aware Chemformer model alone before integrating it in the multistep framework and comparing it to the strategies relying on the broken bonds score (see ESI Results:† Verifying single step disconnection-aware Chemformer). Specifically, the model was evaluated by disconnection matching accuracy, which measures how well the model generates predictions that disconnect the prompted bond to break. The results highlight the ability of the disconnection-aware Chemformer to recognize tagged atoms and generate distinct predictions which break the corresponding bond(s) (Fig. S1 and S2†). We were therefore confident to use the model in the multistep retrosynthesis framework. The model was combined with the template-based model (Table 1), which is the standard single step model in AiZynthFinder^5,32^ (Fig. 1d). The predictions of the two models were simply concatenated and sorted according to the model probabilities associated with each prediction. For technical details on how the two models were integrated in AiZynthFinder, we refer to Methods; *Disconnection-aware Chemformer in multistep retrosynthesis*. The disconnection-aware Chemformer was trained to recognize the tags and disconnect the corresponding bond(s). When the input molecule contained *bonds to break*, those were explicitly tagged and provided to the Chemformer. The output consisted of unique reactants atom-mapped by RXN-mapper^33^ in order to reliably propagate the tagged disconnection sites to the next steps in the synthesis route. The input molecule's atom-mapping was then propagated to the predicted reactants (see ESI Methods:† Chemformer expansion policy for details).

#### Combining approaches into disconnection-aware strategies

In summary, we investigated two approaches to human-guided retrosynthesis: we either modified (1) the search algorithm or (2) the single step model. As an alternative to the standard MCTS with the state score as the single objective, we considered an MO-MCTS with the broken bonds score and the state score as objectives. As noted above, we did not consider MCTS with the broken bonds score as a single objective since solvability and route length are important retrosynthesis aspects captured by the state score. For single step predictions, we considered either the standard template-based model or the disconnection-aware Chemformer framework. Note that the template-based model receives molecular fingerprints as input, which prevents tagging of bonds to break. Therefore, we did not consider a disconnection-aware template-based model here. Moreover, we did not consider the baseline Chemformer (not disconnection-aware) as a single step model since extensive comparisons between the template-based model and baseline Chemformer have been carried out in the literature.^34,35^

Building on the two approaches, we implemented four different disconnection-aware strategies in multistep retrosynthesis:

(1) Route ranking: in AiZynthFinder, the tree search generates a large number of synthesis routes which are ranked in a postprocessing step. After ranking the routes, AiZynthFinder extracts the top 10–15 routes which are returned to the user. The number of routes generated by MCTS is much larger (on average ∼125 with the state score), and therefore the score used to rank routes greatly influences which routes are returned to the user. In the route ranking strategy, routes are ranked prior to extraction using a linear combination (with equal weights) of the broken bonds score (Methods; eqn (1)) and the state score. The score was thus not used in the MCTS, but in the subsequent route extraction phase by AiZynthFinder to gather the output routes. This is in contrast to the standard strategy, where only the state score is used for route ranking. Note that the template-based model and standard single-objective (state score) MCTS were used in this strategy.

(2) MO search: multi-objective MCTS^23,24^ (MO-MCTS) with the broken bonds score as an additional objective together with the state score. The template-based model was used as single-step model. The template-based model generated 50 predictions. The broken bonds score ensured that routes with desired disconnections were favored. The two objectives used in the MO-MCTS were used to rank and select routes, such that the extracted routes belonged to the Pareto front.

(3) Chemformer: disconnection-aware Chemformer paired with the template-based model in a multi-expansion framework to generate single step predictions. The standard MCTS with the state score was used to conduct the search. The state score was used to rank and extract routes.

(4) Chemformer-MO: MO-MCTS with the state score and broken bonds score to conduct the search, and the disconnection-aware Chemformer paired with the template-based model in a multi-expansion framework to generate single step predictions. The two scores used in the MO-MCTS were used to rank and select routes, such that the extracted routes belonged to the Pareto front.

The frozen bonds filter was used in all four strategies. The usefulness of the frozen bonds filter is specifically analyzed in the section Application to a target-specific set of compounds. The four disconnection-aware strategies were compared to the standard AiZynthFinder search that uses the template-based model from the production platform and MCTS with the state score as the single search and route ranking objective.^18^

### Performance of breaking bond strategies in multistep retrosynthesis

**Fig. 2:**
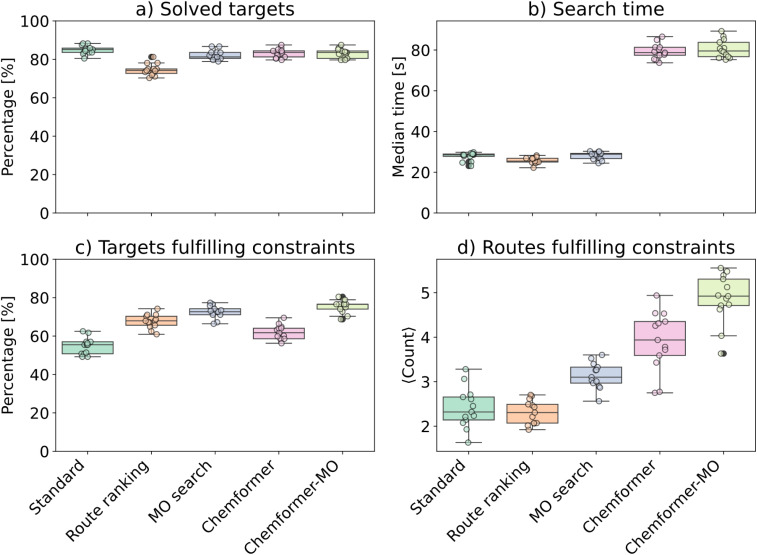
Performance of the different disconnection-aware strategies with standard search as the baseline. Performance is measured in terms of (a) percentage of solved targets, (b) median search time, (c) percentage of targets which are solved and fulfill the bond constraints and (d) average number of routes which are solved and fulfill the bond constraints. The full PaRoutes set-n1 targets were divided into batches with batch size 128 in order to facilitate statistics calculations (Methods; “Statistical variation from batches”). Each colored dot corresponds to one batch. Box-plot elements: center line is the median, box limits are the upper and lower quartiles, whiskers are 1.5 times the interquartile range, and outliers are depicted as black dots.

To evaluate the different disconnection-aware strategies, we analyzed targets from PaRoutes set-n1 and Reaxys-JMC synthetic route datasets. Bond constraints were extracted from the synthetic routes using a convergent disconnection score^36^ (see the Methods section for details). The template-based model returned the 50 highest ranked predicted templates. Because the disconnection matching accuracy saturated at top-5 (Fig. S1†), Chemformer used a beam size of five in the search. Fig. 2 shows the performance of the four breaking bond strategies and standard search on the PaRoutes set-n1 dataset. The two constraint satisfaction metrics were computed on the top (1 to 50) extracted routes (single-objective), or the routes in the extracted Pareto front (multi-objective). Fig. S3† shows the corresponding results for Reaxys-JMC targets.

We could draw several interesting conclusions from the results in Fig. 2. Although the standard search solved most targets (84.91%) in a short time (Fig. 2a and b), it only solved and satisfied the bond constraints for 54.80% of the targets. The Chemformer strategy was substantially slower than the standard strategy, while the route-ranking and multi-objective search strategies were comparable in search time (Fig. 2b). The route-ranking strategy led to fewer solved targets than the standard search (Fig. 2a: 74.62% *vs.* 84.91%) but resulted in more solved targets which fulfill the bond constraints (67.66% *vs.* 54.80%).

The Chemformer strategy yielded almost as many solved targets as the standard strategy (Fig. 2a; 83.18%), while more of the solved targets actually complied with the bond constraints (Fig. 2c; 62.00% *vs.* 54.80%). Although the Chemformer strategy improved bond constraint performance compared to standard search, it remained less efficient than the route-ranking strategy. Hence, the route scoring in the AiZynthFinder postprocessing step is an important, but easily overlooked, component. In contrast to Chemformer, which approaches the problem with single step predictions, strategies that rely on the broken bonds score consider the full composition of the routes. In other words, Chemformer operates locally while the broken bonds score operates globally. The global approach appeared especially successful. In particular, the MO search strategy generated routes which satisfied the constraints for even more targets than the route-ranking strategy (Fig. 2c). Moreover, it generated a larger number of solved routes which fulfilled bond constraints compared to the route-ranking strategy, while a smaller average number of routes was generated compared to the Chemformer strategy (Fig. 2d). The same was observed for the Reaxys-JMC targets (Fig. S3d†). These results demonstrate a clear advantage of using the broken bonds score from human-guided synthesis planning *via* prompting. Because the local and global approaches treat the problem in fundamentally different ways, one could imagine that the combined approach (Chemformer-MO which combines the MO-MCTS with disconnection-aware Chemformer) is superior. Indeed, for the PaRoutes set-n1 targets, the Chemformer-MO strategy reached the highest percentage of targets fulfilling bond constraints and yielded the largest number of routes which satisfy the bond constraints (Fig. 2).

For the Reaxys-JMC targets, Chemformer-MO performed similar to the MO search strategy in terms of solved targets satisfying constraints, but generated substantially more routes which satisfy the constraints (Fig. S3†). We noticed that 6.0% of the targets were solved with fulfilled constraints using MO search (Table 2: Case 1), but not by Chemformer-MO. Conversely, 4.2% of the targets were solved with fulfilled constraints by Chemformer-MO (Table 2: Case 2), but not by MO search. Because the *bonds to break* constraints were handled differently by these two strategies, we investigated this constraint type. The number of *bonds to break* constraints was typically fewer for targets in Case 2, compared to the targets in Case 1. Moreover, Chemformer-MO often solved routes with fewer steps than the number of constraints in Case 1, as opposed to the MO search in Case 2 (Table 2). While Chemformer-MO did not fulfill all constraints in Case 1, it solved 85% of the targets and satisfied at least one of the *bonds to break* constraints for all targets. This was in stark contrast to the MO search strategy in Case 2 (Table 2: 45%). The premature convergence, which was more common for the Chemformer-based strategy, is a symptom of the *bonds to break* constraints being treated as soft constraints. Because the state score favors routes which are short and solved, the longer routes from Chemformer-MO which satisfied all bond constraints were not included in the Pareto fronts of these targets. With the current implementation, one can include routes from lower Pareto ranks (Table 1) to extract the longer routes that satisfy all bond constraints. Altogether, the results presented here suggest that the combined approach of the Chemformer-MO strategy successfully generated routes which fulfill the given bond constraints, with the largest performance boost originating from the broken bonds score in MO-MCTS.

**Table 2:** Data for Reaxys-JMC targets where either the multi-objective search or the Chemformer-MO strategy (but not both) fulfilled all bond constraints

Case	Fulfilled bond constraints	Distribution of #bonds to break constraints	Solved in less (less or equal to) steps than #bonds to break constraints	Solved targets	Satisfied any bonds to break constraints
1 Fulfilled by MO search (but not Chemformer-MO)	6.0%	1 Constr.: 5%	[Chemformer-MO]: 43% (80%)	[Chemformer-MO]: 85%	[Chemformer-MO]: solved – 85%, all – 100%
		2 Constr.: 48%			
		3 Constr.: 47%			
2 Fulfilled by Chemformer-MO (but not MO search)	4.2%	1 Constr.: 33%	[MO search]: 24% (36%)	[MO search]: 66%	[MO search]: solved – 45%, all – 78%
		2 Constr.: 62%			
		3 Constr.: 8%			

### Characteristics of generated synthesis routes

**Fig. 3:**
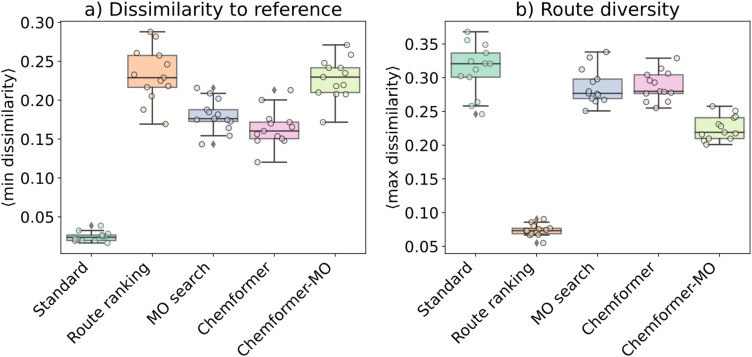
Characteristics of the generated routes from each strategy. (a) Dissimilarity of solved routes compared to the standard search and (b) route diversity of the solved routes in terms of average pairwise route distance. Statistics are gathered from averages over batches of 128 PaRoutes set-n1 targets (Methods; “Statistical variation from batches”). Each colored dot corresponds to one batch. Box-plot (black boxes) elements: center line is the median, box limits are the upper and lower quartiles, and whiskers are 1.5 times the interquartile range.

To characterize the set of suggested routes, we calculated dissimilarity to the routes generated by the standard search, as well as the within-strategy route diversity (Fig. 3). To compute these metrics, we used a recently introduced route similarity metric which reports similarity *via* common bond disconnections and atom groupings across the route.^37^ The dissimilarity to the standard search was measured for each strategy by using the dissimilarity to the most similar standard-search route while diversity was calculated as the average maximum dissimilarity across the set of generated routes. The Methods section includes a detailed explanation of these two metrics.

Fig. 3a (Reaxys-JMC: Fig. S4a†) shows that the disconnection-aware strategies in general suggested dissimilar routes to the standard search. The route-ranking strategy yielded the most dissimilar sets of routes, followed by the Chemformer-MO strategy (Fig. 3a and S4a†). Notably, the dissimilarity of routes when comparing standard search to itself resulted in slight non-zero values. While this might appear unexpected, it is inherent to the used similarity metric.^37^ Specifically, the route similarity consists of two parts: atom-similarity and bond-similarity. The atom-similarity is normalized based on the number of total molecules in the two routes being compared. However, the atom-similarity calculation is only based on atoms that are identified in the final target molecule. This leads to some molecules being completely left out from the similarity calculations while still being part of the normalization. This is sensible when comparing two different routes, but may sometimes lead to noise when comparing two identical routes.

When considering route diversity in terms of average maximum dissimilarity between the set of routes generated by each strategy, the disconnection-aware strategies scored lower than the standard strategy (Fig. 3b and S4b†). In addition to these metrics, we computed the round-trip accuracy of predicted reactions. The round-trip accuracy was similar across the different strategies (Fig. S5†), indicating that any strategy can be employed without excessively compromising route feasibility.

In order to understand how, and to what extent, the disconnection-aware Chemformer was used in the routes, we analyzed the frequency of predictions in the extracted routes which had been generated by Chemformer and Chemformer-MO strategies (Fig. S6 and S7†). The Chemformer expansion policy was used more often in the Chemformer-MO strategy than in the single-objective Chemformer strategy (Fig. S6 and S7†), demonstrating the role of the broken bonds score in steering the search and ranking routes. Regardless of whether single- or multi-objective MCTS was used, Chemformer predictions were found only in the first three steps of the extracted routes (Fig. S6b and S7b†). The depths of trees generated by Chemformer and Chemformer-MO on average only included three steps (Tables S1 and S2†). Thus, the prioritization of disconnecting *bonds to break* left little room for the standard template-based predictions, which were mainly used as a complement to yield properly solved routes. In line with this, we noticed that the template-based model was not utilized in all trees, and was found in fewer trees generated by the Chemformer-MO strategy (Fig. S6 and S7†).

In summary, the disconnection-aware Chemformer produced reliable single step predictions, but the MO-MCTS with the broken bonds score (MO search) had a greater impact on the final constraint satisfaction of generated routes. Furthermore, the combined Chemformer-MO approach exploited features from both strategies and governed several synthesis routes in the feasible region while lowering route diversity compared to the standard search.

### Application to a target-specific set of compounds

To highlight the potential of the overall approach in real-world applications, we analyzed ten molecules originally discovered due to their potency against B lymphoma cells.^38^ We identified two bonds which were common in the ten molecules and prompted the search with these as *bonds to break*: a carbon–nitrogen bond ([N:1]–[c:2]) and an amide bond ([N:3]–[C:4]) (Fig. S8†). These aligned with the SAR (structure activity relation) vectors and allowed us to focus on synthesizing the thiazole core. One of the target molecules did not contain the amide bond. After the retrosynthesis search, we extracted five routes for each strategy.

Table 3 reports the percentage of solved targets and the percentage of targets with routes that were solved with satisfied bond constraints. The standard search reached purchasable precursors for all 10 targets. However, for one of the targets, neither of the three strategies relying solely on the template-based model was able to break the specified bonds (Table 3). In contrast, both Chemformer-based strategies satisfied bond constraints for all targets. Although all strategies performed reasonably well in satisfying bond constraints for this selected set of molecules, the bulk experiments (Fig. 2 and 3) demonstrate clear differences in the overall performance of the disconnection-aware strategies.

**Table 3:** Solvability and constraint compliance of the five strategies on the application dataset of 10 target molecules

Strategy	Solved targets	Fulfilled bond constraints
Standard	10/10	9/10
Route ranking	9/10	9/10
MO search	9/10	9/10
Chemformer	10/10	10/10
Chemformer-MO	10/10	10/10

**Fig. 4:**
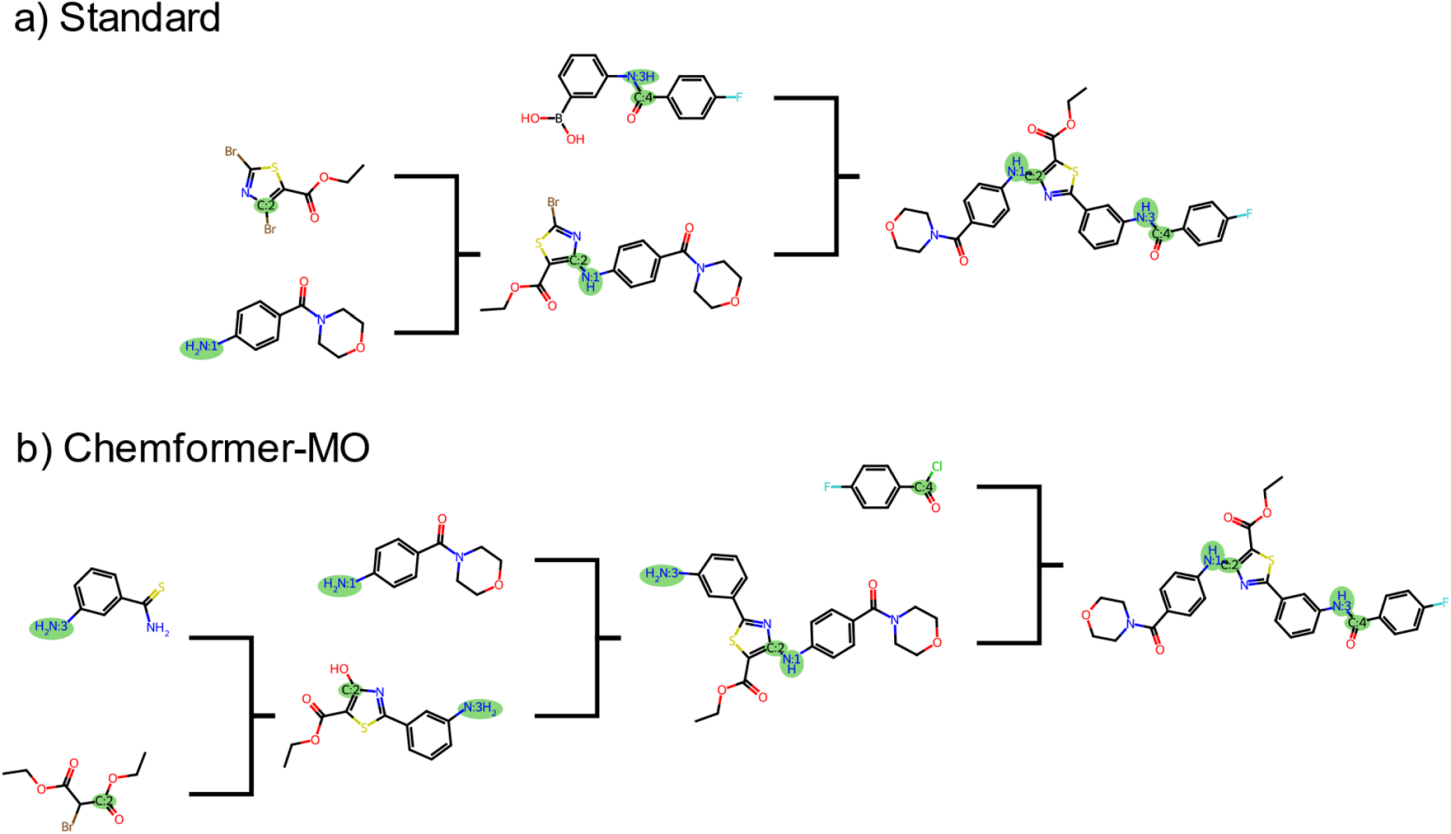
Comparison of the top routes from standard search and the Chemformer-MO strategy. The top routes generated by the (a) standard search and (b) Chemformer-MO strategy for the target for which the standard search did not break the specified bonds. The “bonds to break” are highlighted in green.

Fig. 4 depicts the top-1 routes generated by the standard search and Chemformer-MO strategy for the target for which the template-based strategies did not yield solved routes with fulfilled constraints. Specifically, the route generated with the standard search reached purchasable precursors before breaking the bond [N:3]–[C:4] (Fig. 4a). In practice, one could envision a scenario where the chemist wishes to break a specific bond in order to avoid a certain starting material, for example due to cost or environmental impact. Then, it is important that the prompts made by the chemist are respected by the synthesis planning tool. For this reason, the route generated by the standard strategy in Fig. 4a can be considered less successful compared to the route in Fig. 4b generated by the Chemformer-MO strategy.

To test the effect of the frozen bonds filter, we carried out a search with *bonds to freeze* set to the bond between thiazole and benzene that was disconnected in the first Suzuki coupling reaction (Fig. 4a). With this filter, the bonds to break constraints were satisfied for all targets (Table S3†). However, the routes were not ranked to favor constraint satisfaction, leading to only 7/10 top-1 routes fulfilling the constraints. Together with the route-ranking strategy, the frozen bonds strategy yielded lower route diversity than any of the other strategies. Moreover, the frozen bonds strategy lead to routes more dissimilar to the standard search while the disconnection-aware strategies had higher top-1 constraint satisfactions (Table S3†). As mentioned, the ability to prioritize and rank routes based on constraint satisfaction is an important aspect when constructing routes with common intermediates. In contrast to using a frozen bonds filter, the Chemformer-MO strategy avoided the premature convergence-issue by first breaking the bond [N:3]–[C:4] in the first step with an *N*-acylation to amide reaction and then the bond [N:1]–[c:2] in the second step with nucleophilic aromatic substitution (SNAr)‡‡A triflate or tosylate would be needed to activate the oxygen and make a viable leaving group.§§The thiazole cyclization reaction would benefit from a protecting group on the nitrogen, for example a butyloxycarbonyl (BOC) group. (Fig. 4b). This particular approach was observed in routes suggested by both Chemformer strategies for 7 of the 9 targets which included both bonds.

**Fig. 5:**
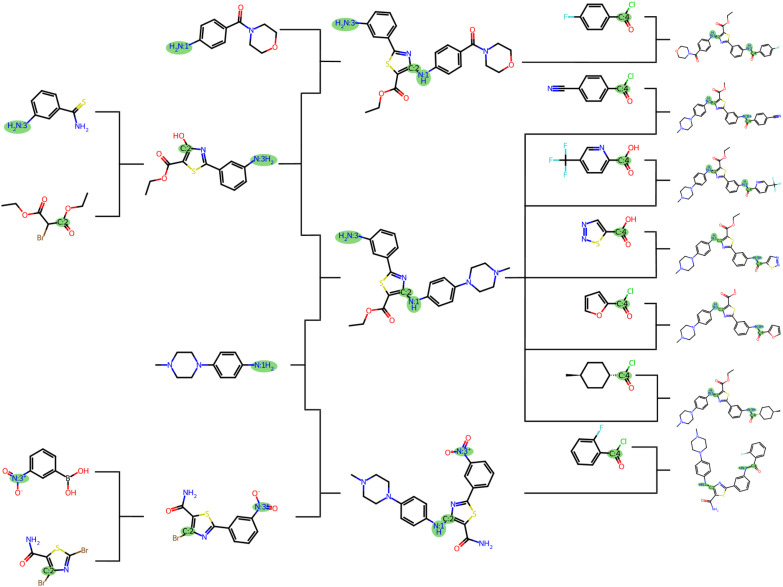
Joint reaction tree for application target molecules. The tree includes targets that obtained routes with the common retrosynthesis approach (breaking bond [N:3]–[C:4] in the first step and [N:1]–[c:2] in the second). The “bonds to break” are highlighted in green.

For the seven targets which shared a common retrosynthesis approach where bond [N:3]–[C:4] was disconnected in the first step and [N:1]–[c:2] in the second, we constructed a joint reaction tree (Fig. 5). The routes presented in this figure correspond to the top route of each target molecule. Five of these targets shared a common intermediate at the second step, and six shared a common intermediate at the third step. Interestingly, the shared intermediate at the second step was the target molecule which only contains the bond [N:1]–[c:2] (Fig. S8†). Hence, 8 of the 10 targets are actually represented in the joint reaction tree. One target did not share any intermediate molecules with the other targets, but a building block molecule which was common to the five targets with a common second-step intermediate.^3^¶¶The route is missing a step of reducing the nitroarene before transforming into an amide. Altogether, the Chemformer-MO strategy generated routes with common intermediates and shared building block molecules, greatly simplifying the synthesis of these compounds. For this set of compounds, the local approach used by the disconnection-aware Chemformer thus proved successful when the template-based model did not generate routes where [N:3]–[C:4] was disconnected in the first step.

## Conclusions

We have developed and benchmarked a novel approach for human-guided retrosynthesis *via* bond prompting. In the current AiZynthFinder framework, the user can provide bonds to break and bonds which should be frozen throughout the search. To prevent bonds from breaking, we implemented a filter which immediately discards those reactions that break one of the *bonds to freeze*. For *bonds to break*, we benchmarked four disconnection-aware strategies that either relied on a novel broken bonds score, a disconnection-aware Chemformer tailored for multistep retrosynthesis, or both.

We demonstrated that the strategies relying on a broken bonds score (route-ranking and MO search) overall outperformed the basic Chemformer strategy. Because the template-based model returned the 50 highest ranked template predictions, the bond to break was often disconnected in one of these. The broken bonds score could then rank the reaction trees or steer the search to generate routes which fulfilled the given bond constraints. In the Chemformer strategy, the boosted single step predictions were likely down-prioritized because the default search objective favored short and solved routes, which may be suboptimal for this task. When combining the disconnection aware Chemformer with the broken bonds score in a multi-objective search, features from both local and global approaches were exploited, leading to a higher percentage of solved routes with satisfied constraints compared to the other three strategies. Notably, the Chemformer and Chemformer-MO strategies were significantly slower than standard search, while the MO search and route-ranking performed on par with the standard search concerning search time. Therefore, the strategies only relying on the broken bonds score and the template-based model are suitable in industrial application settings where multiple searches might be conducted, and time is a limiting factor. Conversely, the Chemformer-MO strategy may be better suited for few-target searches where the project can afford to generate more suggestions of routes which comply with the bond constraints at the cost of computational time.

The work presented here can be exploited in several practical application scenarios. For example, given the knowledge and experience of a chemist, human-guided retrosynthesis can refine the synthesis routes provided by a standard search where undesirable disconnections have been proposed. Another example is synthesis planning for a set of similar molecules, for instance those obtained from generative molecular design with reinforcement learning.^39^ Adopting our approach may in these cases improve the routes, thus simplifying the synthesis process and consequently accelerating drug discovery.

In conclusion, the disconnection-aware Chemformer with the multi-objective search strategy used together with a frozen bonds filter is a viable strategy to generate routes that comply with user-specified bond constraints, and enables creating joint synthesis plans for a compound series using human knowledge. Future work includes creating a complete workflow for multi-target synthesis which integrates our approach into existing multi-target planning framework tools.^21,22,29^

## Methods

### Broken bonds score

To rank routes during and after the retrosynthesis search, we devised a novel score, the broken bonds score. The broken bonds score returns a value between zero and one and gives a higher score to routes which disconnect bonds to break earlier in the tree, and a lower score to the routes which do not satisfy these constraints.

The score takes a set of bonds to break, 
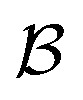
, as the input and returns a score, 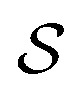, between zero and one:1
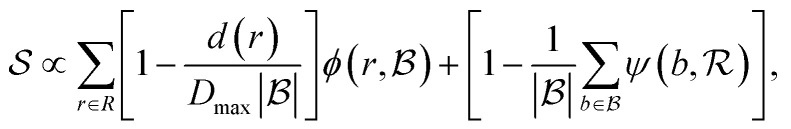

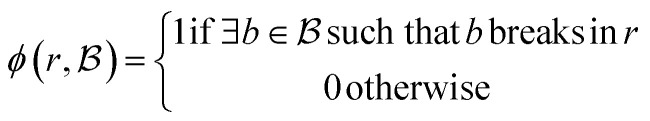

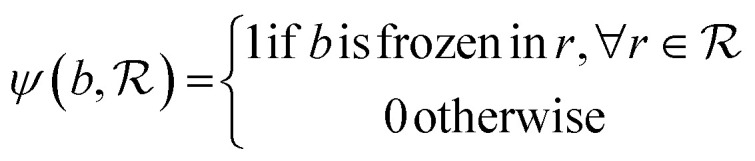
Here, *d*(*r*) is the depth of reaction *r*, which breaks some bond *b*, in the reaction tree. Furthermore, 
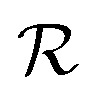
 is the set of reactions in the reaction tree and *D*_max_ is the maximum depth of the tree. The reaction depth found in the first component ensures that breaking a bond early in the search tree gives a higher score than a bond being disconnected further down in the tree. The second component represents unbroken bonds which are given a joint penalty corresponding to breaking at the maximum depth, *D*_max_. Note that the score is normalized with the number of productive components (
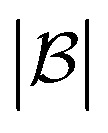
 if all bonds are broken or 
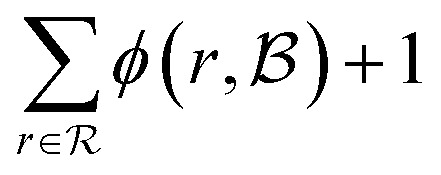
 if not all bonds are broken). If all bonds to break are disconnected in the first reaction, the score becomes 
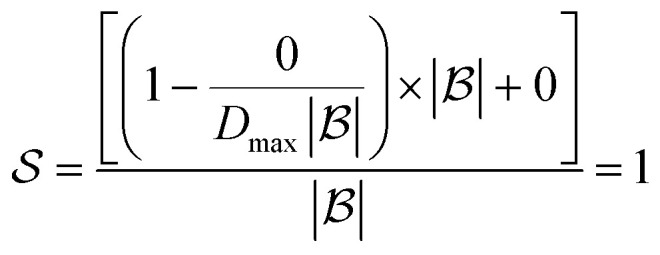
. Conversely, if none of the bonds to break are disconnected, the score becomes 
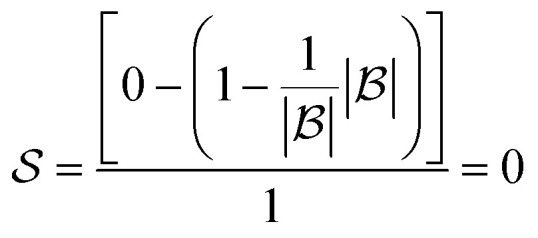
. Finally, the score for the third reaction tree in Fig. 1c is calculated as 
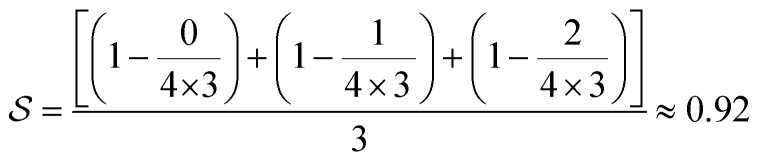
.

### Disconnection-aware Chemformer

#### Datasets for single step Chemformer

The single step Chemformer was trained and evaluated on the open source USPTO-50k^40^ and a curated proprietary dataset from AstraZeneca which contains 17.4 million reactions. The template-based model used in multistep retrosynthesis with AiZynthFinder^41^ was trained on the proprietary dataset as well.^32^ USPTO-50k is a common and publicly available benchmark dataset for retrosynthesis models, while the proprietary dataset spans a larger chemical space and is more suitable for the purpose of drug discovery. The proprietary dataset was divided into training (90%), validation (5%), and test (5%) sets. Furthermore, reactions from PaRoutes^42^ were placed in the test set. For USPTO-50k, we used the dataset split already published in the original Chemformer study.^30^ The data preprocessing was performed with the AiZynthTrain tool.^41^

#### Chemformer training and validation

We fine-tuned a disconnection-aware Chemformer. To validate the disconnection-aware model for single step predictions, we additionally fine-tuned a baseline Chemformer. The baseline model was thus not used in the multistep retrosynthesis experiments. The two models were fine-tuned on both the USPTO-50k^40^ and the proprietary dataset. The baseline backward model predicts reactants given (an unmodified) product SMILES as the input. In the disconnection aware Chemformer, the bond to break is marked by tagging the corresponding atoms with “<atom>!”, as suggested in previous work.^25^ The data preprocessing tools developed here are now available in AiZynthTrain.^41^ In addition to the backward models, we fine-tuned a forward Chemformer model on the proprietary dataset for round-trip validation of backward model predictions. The fine-tuning followed the approach reported in earlier work.^30,34^ See ESI Methods† for more details on dataset preparation, fine-tuning and validation of the predictions produced by disconnection-aware Chemformer.

#### Disconnection-aware Chemformer in multistep retrosynthesis

To incorporate the disconnection-aware Chemformer in the AiZynthFinder multistep synthesis planning tool, we developed a novel framework which included a multi-expansion strategy. The multi-expansion policy combines the output of multiple expansion strategies (Table 1), in this case the expansion policy of the standard AiZynthFinder single step model and the (disconnection-aware) Chemformer expansion policy. The latter is referred to as the Chemformer expansion policy and is described in the next paragraph. The standard single step model in AiZynthFinder is a template-based model; a neural network which ranks a set of reaction templates given the fingerprint of a target molecule.^5,32^ The template-based model was applied in all iterations, while the disconnection-aware Chemformer was only applied whenever the input molecule contained the bonds to break. The predictions of the two expansion strategies were concatenated and sorted according to the priors (model output probabilities) associated with each prediction. Because the multi-expansion strategy combined the template-based and disconnection-aware predictions based on their priors, both models could contribute to the suggestions for the next step in the search. The priors of the two models were weighted equally. This resulted in Chemformer predictions often being ranked higher than the template-based model, but also allowed for mixing of the predictions (Fig. S9†).

The Chemformer expansion policy received an atom-mapped SMILES as input together with the list of bonds to break. In an atom-mapped SMILES, each atom is tagged by a number. Atom-mapping is used to pair the corresponding atoms in product and reactants SMILES in a reaction. By exploiting the atom-mapping predicted by RXN-mapper,^33^ the original atom-mapping could be propagated to the retro-reactions predicted by Chemformer. The Chemformer expansion policy returned atom-mapped predictions which break the bonds specified by the list of bonds to break. The technical details of data processing, including how the atom-mapping is propagated in each step, and filtering carried out in the Chemformer expansion policy are given in ESI Methods:† Chemformer expansion policy.

Similar to when the template-based model was used alone, the multi-expansion strategy returned the first ranked 50 template predictions of the two combined models to the tree search.

### Multistep retrosynthesis experiments

#### Synthetic datasets

Multistep retrosynthesis searches were carried out on compounds from PaRoutes set-n1 (ref. 42 and 43) and Reaxys-JMC. Moreover, we used eMolecules as the stock.^44^ The sources for Reaxys-JMC were extracted from the *Journal of Medicinal Chemistry* between the years 2000 to 2020 as deposited in the Reaxys database.^28^ The two datasets contained complete routes and could therefore be used to construct ground truth data for multistep retrosynthesis. Ground truth routes were constructed by first grouping by citation and then creating reaction networks from which routes could be extracted with an in depth first search.^28^ The approach for building synthetic routes has been detailed elsewhere.^42,45^ We retrieved 2500 randomly sampled (without replacement) targets from PaRoutes set-n1, using numpy's random.choice function and seed set to 1. Moreover, we extracted ∼1600 targets from Reaxys-JMC which were not part of the Chemformer training set. Because the reactions in the PaRoutes set-n1 dataset are public and had been completely excluded from both single step models' training sets, we used this dataset as the main data, while the Reaxys-JMC results were used for validating the findings.

To create synthetic benchmarking datasets for the disconnection-aware multistep retrosynthesis, we first extracted all reactions from the reference routes (PaRoutes and Reaxys-JMC). From these reactions, we extracted bonds to break and bonds to freeze with a workflow which included propagating atom-mapping in the tree and selecting bonds to break by maximizing the convergent disconnection score.^36^ The convergent disconnection score makes sure that we select bonds that split the product molecule into roughly equal sized reactants. The workflow for obtaining bonds to break and bonds to freeze for a target molecule from an example route is visualized in Fig. S10,† and described in detail in ESI Methods:† Synthetic benchmarking datasets. The bonds to freeze were selected from the unchanged bonds to ensure that there is at least one possible solved route (the reference route) for each target molecule. We note that the freezing some of the changed bonds in the reference routes would force the search to find different solved routes than the reference but would not guarantee an existing solution. In addition, we chose to freeze fewer bonds than bonds to break to focus on the effect of the different disconnection strategies, and to lower the probability of freezing necessary bonds for alternative routes. Applying this bond extraction workflow resulted in 1748 and 1367 targets from PaRoutes set-n1 and Reaxys-JMC, respectively.

#### MCTS hyperparameters

We used the default AiZynthFinder hyperparameter values for maximum search tree depth, search tree width and number of iterations, as these were identified in a data-driven fashion and have been shown to give an optimal balance between median search time and percentage of solved targets.^46^ To provide a fair comparison between template-based and Chemformer strategies, we set the maximum search time to 300 s, regardless of the search strategy. In other words, the MCTS stops when succeeding 300 s. We refer to our recent study on MCTS hyperparameters^46^ for more details on how the hyperparameters affect the search.

#### Evaluation metrics

Multistep retrosynthesis predictions were evaluated based on the search time and percentage of solved targets. In addition to this, we specified a few custom breaking bond metrics: (1) the percentage of targets which were solved and satisfied all bond constraints (bonds to freeze as well as bonds to break), and (2) the average number of trees which satisfied bond constraints. To verify the reactions predicted in the search, the top-10 round-trip accuracy was computed. Round-trip top-N accuracy validates the predictions using a forward Chemformer model trained to predict products given reactants as input. More details are found in ESI Methods.† Furthermore, we evaluated the generated routes in terms of dissimilarity to the routes generated by the standard search, and route diversity. To compute these metrics, we used a recently introduced similarity metric^37^ which calculates similarity between 0 and 1 in terms of disconnected bonds and grouping of atoms in the routes. Dissimilarity, *γ*, to standard search routes was computed as *γ* = 1 − *ζ*_max_, where *ζ*_max_ is the average maximum similarity between each route and the routes in the reference set. This metric thus represents the average dissimilarity of each route and their most similar route generated by the standard search. Route diversity was calculated similarly with *κ* = 1 − *ζ*_min_, where *ζ*_min_ is the average minimum similarity to the routes within the set of generated routes. This metric represents the average dissimilarity of each route and their least similar route in the set of generated routes.

#### Statistical variation from batches

The metrics were evaluated on batches (groups) of target molecules to facilitate statistics calculations. In this case, each batch contained 128 molecules. Metrics, such as search time, typically vary between target molecules. The statistical variation between batches thus represents the uncertainty of an aggregated metric (mean, median. and accuracy) for each batch.

## Software availability

AiZynthFinder with MO-MCTS and broken bonds score was used to run multistep experiments and can be found at: https://github.com/MolecularAI/aizynthfinder. The disconnection-aware Chemformer to run with AiZynthFinder can be found at: https://github.com/MolecularAI/chemformer. AiZynthTrain was used to tag disconnection-sites in the Chemformer training data and can be found at: https://github.com/MolecularAI/aizynthtrain. The LSTM used for computing approximate route distances can be found at: https://github.com/MolecularAI/route-distances. A demo for running the disconnection-aware strategies on the application data using publicly available models and code is available here: https://zenodo.org/records/13626786.

## Author contributions

A. M. W. and S. G. contributed to project conceptualization. A. M. W. developed and implemented the disconnection-aware Chemformer. L. S. developed and implemented the broken bonds score. S. G. and A. M. W. performed the analysis of the application dataset. L. S., A. M. W and S. G. reviewed the software. A. M. W. conducted the retrosynthesis experiments and corresponding analysis. A. M. W. wrote the first draft of the paper. All authors contributed to the writing and manuscript revision.

## Conflicts of interest

The authors are employed at AstraZeneca R&D.

## Supplementary Material

SC-016-D5SC00927H-s001

## Data Availability

The study was mainly carried out using the PaRoutes dataset^43^ (https://github.com/MolecularAI/PaRoutes) and validated by the proprietary Reaxys-JMC, which cannot be shared. The training and evaluation of the disconnection-aware Chemformer was done with USPTO-50k (the original dataset with split: https://github.com/MolecularAI/Chemformer) and the proprietary dataset from AstraZeneca, which cannot be shared. The USPTO-50k disconnection-aware Chemformer and data for the application case are available here: https://zenodo.org/records/13626786.
