# Supplementary material for: Human-guided synthesis planning via prompting
Source: Chem Sci. 2025 Jul 14;16(32):14655–67. doi: 10.1039/d5sc00927h (PMC12258405; doi:10.1039/d5sc00927h)
Supplement: SC-016-D5SC00927H-s001 [file SC-016-D5SC00927H-s001.pdf]

Supplementary Information (SI) for Chemical Science.
This journal is © The Royal Society of Chemistry 2025

Supplementary Information

Supplementary Methods

Disconnection-aware Chemformer

Dataset preparation The input to the disconnection-aware Chemformer is a modified SMILES string, where the *bond to break* is marked by tagging the corresponding atoms with "<atom>!". To create the training dataset, atom-mapped reaction SMILES were used to identify the atoms whose environments change[1]. These atoms were first tagged with "[<atom>:1]" and then converted into "<atom>!" to reduce the required vocabulary[2]. The conversion was done by tokenizing the canonicalized tagged SMILES (as defined by the unmapped SMILES), and then replacing each tagged token with the corresponding original SMILES token, followed by an exclamation mark. The implementation for tagging is available in AiZynthTrain[3] and rxnutils[4].

Fine-tuning Chemformer models We fine-tuned 1) a baseline (backward) Chemformer and 2) a disconnection-aware Chemformer on both the proprietary dataset and USPTO-50k. The baseline backward models predict reactants given (untagged) product SMILES as input. In addition, we fine-tuned a forward Chemformer model on the proprietary dataset for round-trip validation of backward model predictions (see the following paragraph). For USPTO-50k, the Chemformer available from an earlier study[5] was used as baseline. The fine-tuning of the other models followed the approach reported in earlier work[5,6]. The training was done with a batch size of 64, and the baseline backward and forward Chemformers were trained on proprietary data for 30 epochs. The baseline Chemformers were used as pre-trained models to fine-tune the disconnection-aware models. The USPTO-50k model was further fine-tuned for 35 epochs, and the proprietary model for 15 epochs. The Adam optimizer[7] was used together with an augmentation probability of 0.5 and 0.0, as well as a learning rate of 0.001 and 0.0005 for the baseline and disconnection-aware models, respectively. The models trained on the proprietary data, as well as the disconnection-aware USPTO-50k-Chemformer used an updated vocabulary which included the token "!".

Validating single step predictions Each disconnection-aware Chemformer was compared to the corresponding baseline Chemformer. The evaluation metrics included 1) exact matching, 2) round-trip and 3) disconnection top-N accuracy. The exact matching accuracy measures the ability of a model to recover the ground-truth reactants in the dataset. Because multiple sets of reactants may be combined to form the same product, we also computed round-trip top-N matching accuracy. Round-trip top-N accuracy validates the predictions using a forward Chemformer model trained to predict products given reactants as input[8,9]. While the forward model is more specific and can validate more predictions, it is noteworthy that the round-trip accuracy becomes dependent on the forward model performance. For example, the backward Chemformer can extrapolate beyond the training set to create novel reactions. The forward model may fail to validate such novel reactions, even if those are indeed valid[6]. In addition, the round-trip accuracy does not entail whether the *bond to break* was disconnected. We therefore also computed the disconnection top-N matching accuracy[1]. This accuracy was obtained by tagging disconnection sites in the products given the predicted reactions, and comparing the tagged product with the ground-truth dataset. Note that round-trip and disconnection top-N accuracies are calculated in the

same way as top-K exact matching accuracy. The round-trip top-N accuracy used here is often referred to as *coverage* elsewhere[8].

Chemformer expansion policy

The (disconnection-aware) Chemformer expansion policy received an atom-mapped SMILES as input together with the list of *bonds to break*. The atom-mapping was removed for all atoms which were not in the list of *bonds to break*, while atoms which were in the list were tagged by "[<atom>:1]". The atom-map tagging was converted to "<atom>!". The predicted reactants were deduplicated, and invalid molecules were removed. Moreover, the predicted reactions were atom-mapped with RXN-mapper[10] to propagate atom-mapping in the MCTS. The output consisted of the predicted (atom-mapped) reactants, as well as the product with the new atom-mapping. The product with the new atom-mapping was used in AiZynthFinder to propagate the original atom-mapping to the predicted reactants with substructure matching to the product with the input atom-mapping. Finally, predictions which did not disconnect the input bond were removed. The Chemformer log-likelihoods were rescaled with SoftMax.

Synthetic benchmarking datasets

To create the synthetic benchmarking datasets for the disconnection-aware multistep retrosynthesis, we first extracted all reactions from the reference routes (PaRoutes and Reaxys-JMC). The workflow for obtaining bonds to break and bonds to freeze for a target molecule from an example route is visualized in Figure S10. Atom-mapping of each reaction was obtained with RXN-mapper[10]. The atom-mapping from the parent reaction was propagated to the reactants in the following reaction in order to keep the same atom-mapping throughout the reaction tree. For each re-mapped reaction, we identified the disconnection sites in the product. To obtain bonds to break for each target, we applied the convergent disconnection score[11] and kept at most three disconnected bonds which had the highest positive convergence score. We then randomly chose $N_{bonds} - 1$ bonds to freeze from the set of bonds with unchanged atomic environments in the route, where N_{bonds} is the number of bonds to break.

Supplementary Results

Verifying single step disconnection-aware Chemformer

The disconnection-aware Chemformer was compared with the baseline Chemformer on both USPTO-50k and a proprietary dataset. The performance was measured by exact, round-trip and disconnection matching accuracy (Figure S1). In line with previous findings[1], both models achieved high round-trip accuracies with median top-10 above 0.8, while the disconnection-aware model showed improved exact and disconnection-site matching accuracies. The disconnection-site matching accuracies were higher than the exact matching accuracy (Figure S1ii: top-1: 0.85 vs. 0.62, top-10: 0.92 vs. 0.85), indicating that the model generated alternative disconnections to those in the ground truth dataset. Furthermore, the disconnection-aware and baseline models generated similar number of valid SMILES, while the disconnection-aware model sampled a larger fraction of unique SMILES (Figure S2).

Supplementary figures

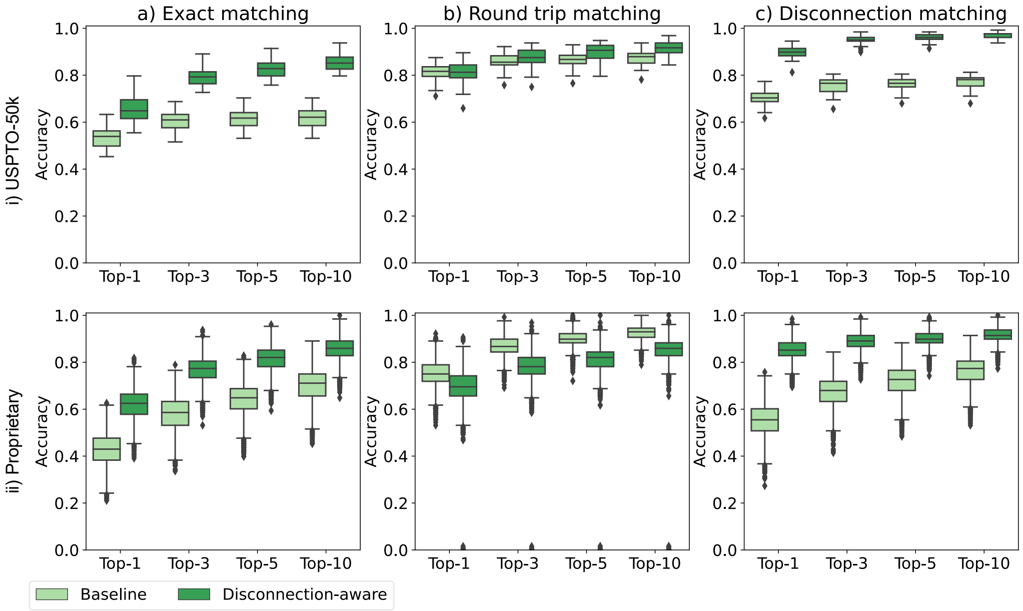

Figure S1 *Top-N accuracy of single step predictions of Chemformer models trained and evaluated on i) USPTO-50k and ii) Proprietary data. Top-N accuracy is reported in terms of a) exact matching to ground-truth reactants, b) round-trip matching using Chemformer forward, and c) disconnection-site matching. Light green corresponds to the baseline Chemformer while dark green is the disconnection-aware Chemformer. Box-plot elements: center line is the median, box limits are the upper and lower quartiles, whiskers are 1.5 times the interquartile range, outliers are depicted as black dots.*

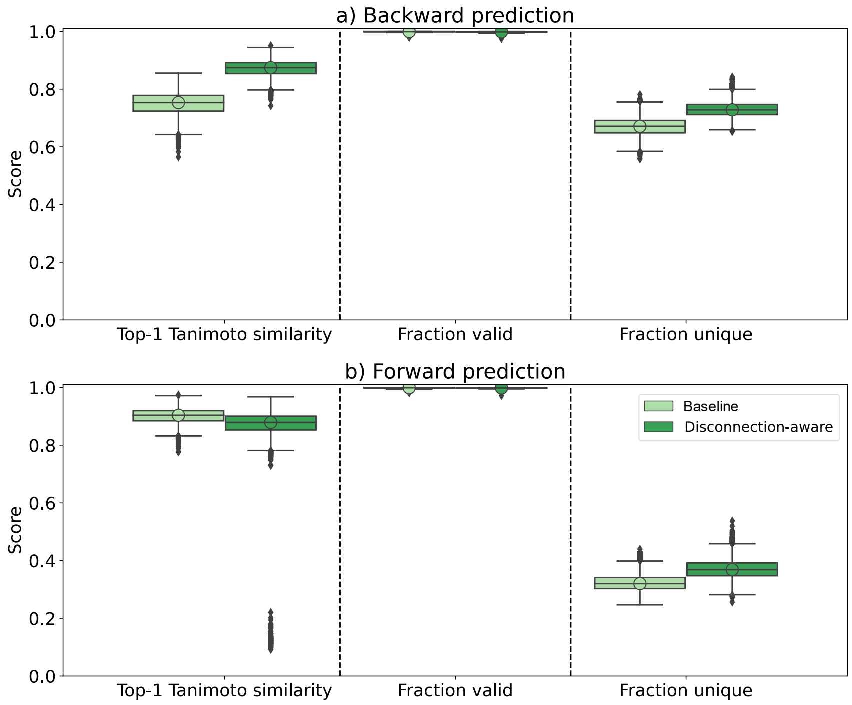

Figure S2 *Inference metrics of Chemformers (baseline – light green, disconnection-aware – dark green) on the proprietary dataset. The metrics were obtained during a) backward (retrosynthesis prediction) and b) forward (round-trip) inference. Box-plot elements: center line is the median, box limits are the upper and lower quartiles, whiskers are 1.5 times the interquartile range, outliers are depicted as black dots.*

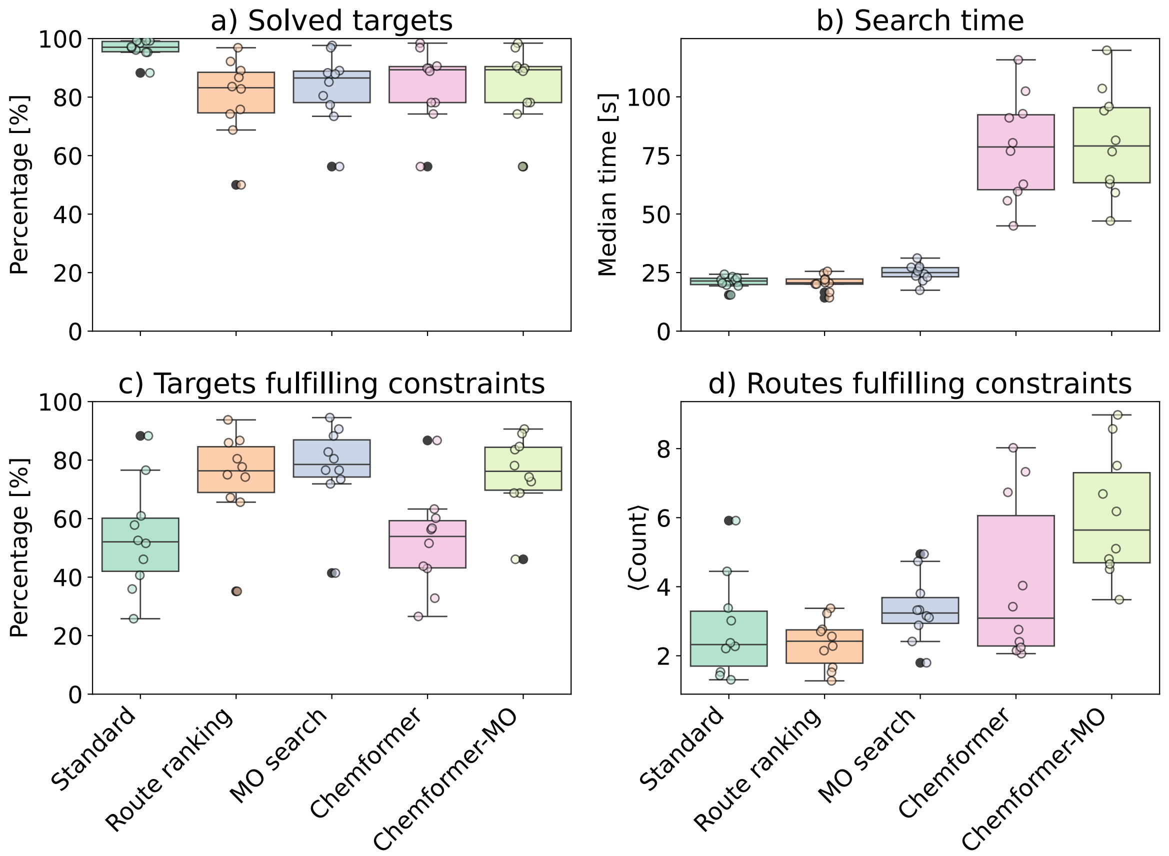

Figure S3 *Performance of the different disconnection-aware strategies with standard search as baseline. Performance is measured in terms of a) percentage of solved targets, b) median search time, c) percentage of targets which were solved and satisfy the bond constraints and d) average number of routes which were solved and satisfy the bond constraints. The statistics were computed over batches of 128 Reaxys-JMC targets. Each colored dot corresponds to one batch. Box-plot elements: center line is the median, box limits are the upper and lower quartiles, whiskers are 1.5 times the interquartile range, outliers are depicted as black dots.*

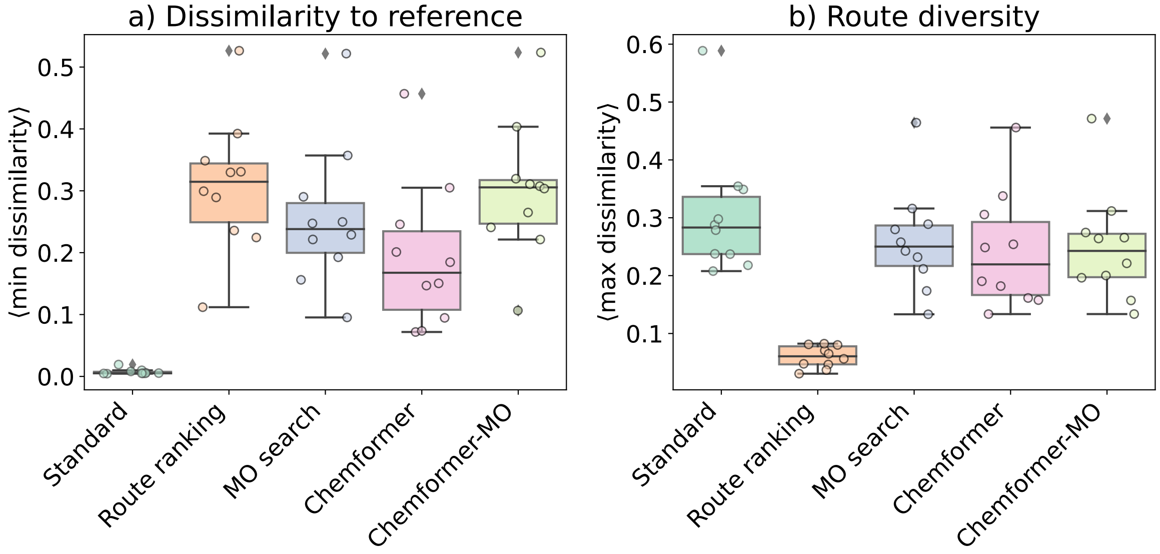

Figure S4 *a) Dissimilarity of generated routes compared to the standard strategy and b) route diversity of the generated routes in terms of average pairwise route distance. Statistics were gathered from averages over batches of 128 Reaxys-JMC targets. Each colored dot corresponds to one batch. Box-plot (black boxes) elements: center line is the median, box limits are the upper and lower quartiles, whiskers are 1.5 times the interquartile range, outliers are depicted as black dots.*

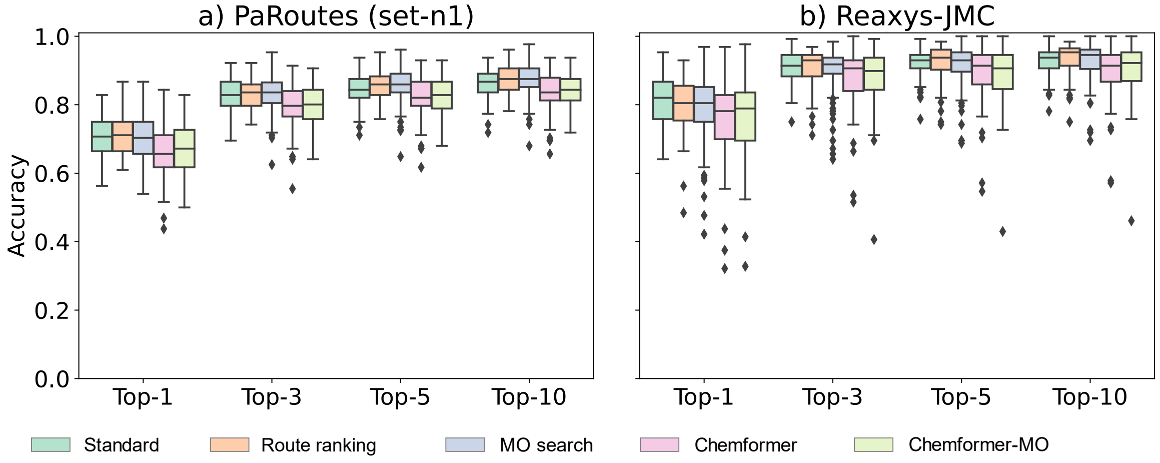

Figure S5 *Round-trip top-N accuracy of reactions extracted from retrosynthesis experiments on a) PaRoutes set-n1 and b) Reaxys-JMC targets. Statistics were computed over batches of 128 samples. Box-plot elements: center line is the median, box limits are the upper and lower quartiles, whiskers are 1.5 times the interquartile range, outliers are depicted as black dots.*

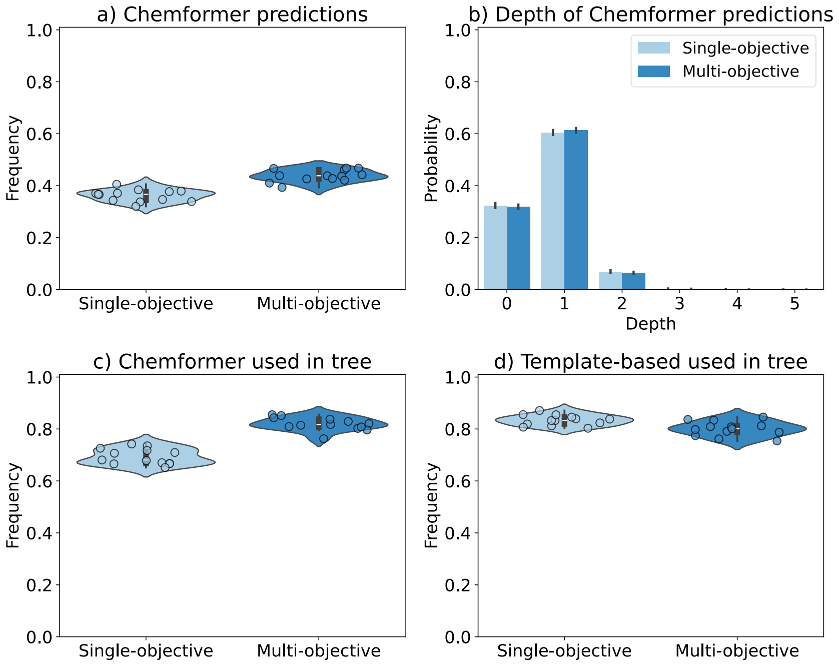

Figure S6 *Expansion policy statistics. a) Frequency of Chemformer expansion policy used in all reactions. b) Occurrence of Chemformer expansion policies at different depths in the reaction trees. c) Frequency of trees with at least one Chemformer reaction (error bars are 95% CI). d) Frequency of trees with template-based expansion policy in at least one reaction. Averages were computed over batches of 128 PaRoutes set-n1 targets in a), c) and d). Each colored dot represents a batch. Box-plot (black boxes) elements: center line is the median, box limits are the upper and lower quartiles, whiskers are 1.5 times the interquartile range, outliers are depicted as black dots.*

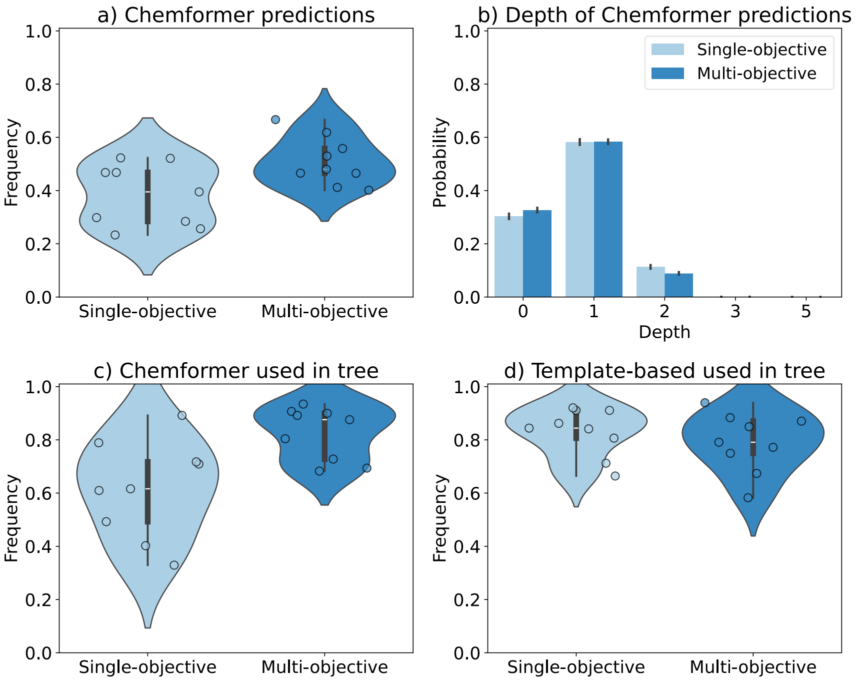

Figure S7 *Expansion policy statistics. a) Frequency of Chemformer expansion policy used in all reactions. b) Occurrence of Chemformer expansion policies at different depths in the reaction trees (error bars are 95% CI). c) Frequency of trees with at least one Chemformer reaction. d) Frequency of trees with template-based expansion policy in at least one reaction. Averages were computed over batches of 128 Reaxys-JMC targets in a), c) and d). Each colored dot represents a batch. Box-plot (black boxes) elements: center line is the median, box limits are the upper and lower quartiles, whiskers are 1.5 times the interquartile range, outliers are depicted as black dots.*

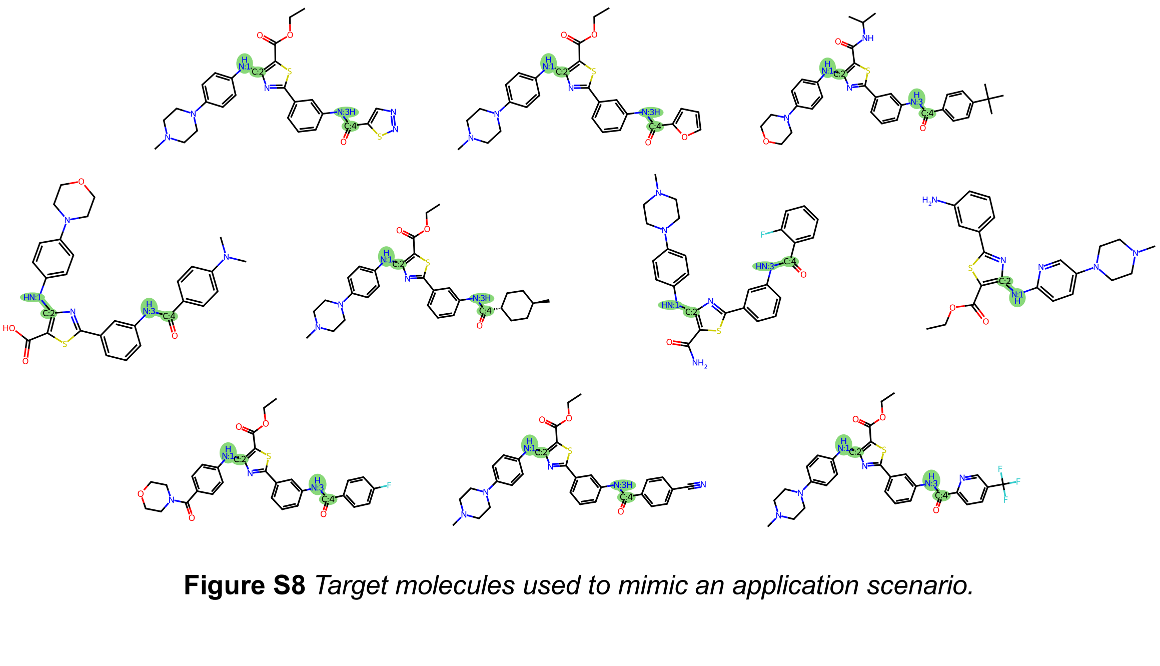

Figure S8 *Target molecules used to mimic an application scenario.*

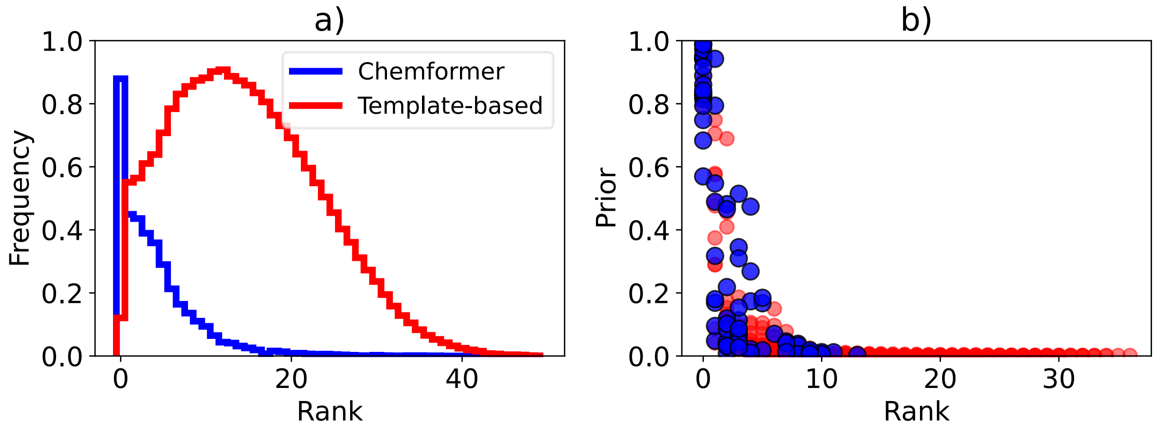

Figure S9 a) *Frequency of Chemformer expansion policy at different ranks. b) Example output showing the priors and policy at each rank for the first 20 compounds. The figures are created from one-step expansion with the disconnection-aware Chemformer and Template-based models using compounds and bonds to break from the PaRoutes set-n1 dataset.*

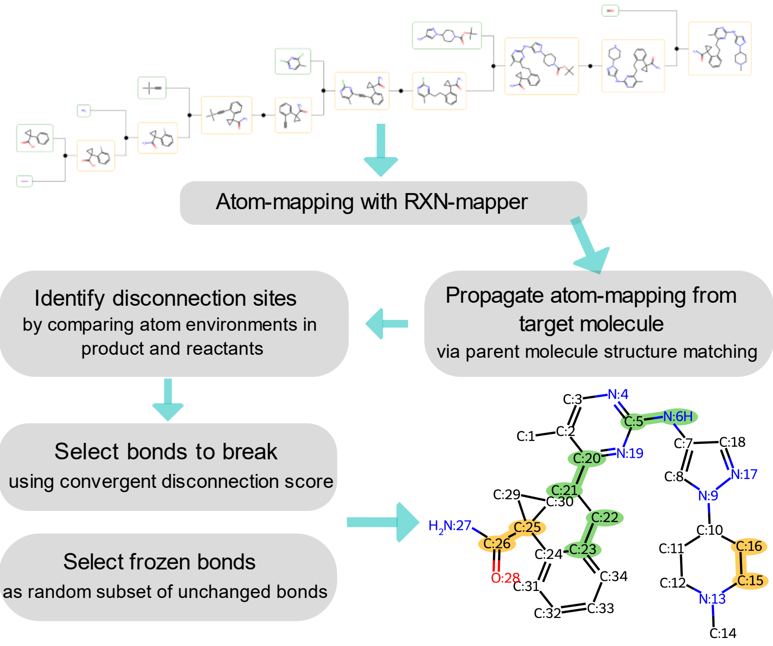

Figure S10 *The workflow for creating multistep retrosynthesis benchmarking datasets. The extracted "bonds to break" (green) and "bonds to freeze" (yellow) are visualized on a target molecule from the PaRoutes set-n1 dataset.*

Supplementary tables

Table S1 *Tree depth statistics (0-indexed) of the disconnection-aware strategies applied to PaRoutes set-n1 targets.*

Strategy	Mean ± standard deviation	median	max
**Standard**	1.97 ± 0.98	2	6
**Route-ranking**	2.59 ± 1.39	2	6
**MO search**	2.75 ± 1.49	2	6
**Chemformer**	2.03 ± 0.94	2	6
**Chemformer-MO**	2.21 ± 0.94	2	6

Table S2 *Tree depth statistics (0-indexed) of the disconnection-aware strategies applied to Reaxys-JMC targets.*

Strategy	Mean ± standard deviation	median	max
**Standard**	1.95 ± 0.73	2	5
**Route-ranking**	2.42 ± 1.08	2	6
**MO search**	2.50 ± 1.30	2	6
**Chemformer**	2.00 ± 0.70	2	6
**Chemformer-MO**	2.09 ± 0.66	2	6

Table S3 *Constraint satisfaction, dissimilarity to reference (standard search) and route diversity. The disconnection-aware strategies are compared to the standard search and freezing the bond between the thiazole and benzene rings.*

Strategy	Solved targets	Fulfilled constraints	Dissimilarity to standard	Route diversity
**Freezing bond**	10/10	10/10 (7/10 top-1)	0.28	0.18
**Standard**	10/10	9/10 (9/10 top-1)	0.00	0.33
**Route ranking**	9/10	9/10 (9/10 top-1)	0.09	0.18
**MO search**	9/10	9/10 (9/10 top-1)	0.08	0.34
**Chemformer**	10/10	10/10 (10/10 top-1)	0.11	0.32
**Chemformer-MO**	10/10	10/10 (10/10 top-1)	0.10	0.38

References

1. Thakkar, A. *et al.* Unbiasing Retrosynthesis Language Models with Disconnection Prompts. *ACS Cent. Sci.* **9**, 1488–1498 (2023).
2. Kreutter, D. & Reymond, J.-L. Multistep retrosynthesis combining a disconnection aware triple transformer loop with a route penalty score guided tree search. *Chem. Sci.* (2023) doi:10.1039/D3SC01604H.
3. Genheden, S., Norrby, P.-O. & Engkvist, O. AiZynthTrain: Robust, Reproducible, and Extensible Pipelines for Training Synthesis Prediction Models. *J. Chem. Inf. Model.* **63**, 1841–1846 (2023).
4. Kannas, C., Thakkar, A., Bjerrum, E. & Genheden, S. rxnutils – A Cheminformatics Python Library for Manipulating Chemical Reaction Data. Preprint at https://doi.org/10.26434/chemrxiv-2022-wt440-v2 (2022).
5. Irwin, R., Dimitriadis, S., He, J. & Bjerrum, E. J. Chemformer: a pre-trained transformer for computational chemistry. *Mach. Learn. Sci. Technol.* **3**, 015022 (2022).
6. Westerlund, A. M. *et al.* Do Chemformers Dream of Organic Matter? Evaluating a Transformer Model for Multistep Retrosynthesis. *J. Chem. Inf. Model.* (2024) doi:10.1021/acs.jcim.3c01685.
7. Kingma, D. P. & Ba, L. J. Adam: A Method for Stochastic Optimization. in (Ithaca, NYarXiv.org, 2015). doi:https://doi.org/10.48550/arXiv.1412.6980.
8. Schwaller, P. *et al.* Predicting retrosynthetic pathways using transformer-based models and a hyper-graph exploration strategy. *Chem. Sci.* **11**, 3316–3325 (2020).
9. Jaume-Santero, F. *et al.* Transformer Performance for Chemical Reactions: Analysis of Different Predictive and Evaluation Scenarios. *J. Chem. Inf. Model.* **63**, 1914–1924 (2023).
10. Schwaller, P., Hoover, B., Reymond, J.-L., Strobelt, H. & Laino, T. Extraction of organic chemistry grammar from unsupervised learning of chemical reactions. *Sci. Adv.* **7**, eabe4166 (2021).

11. Ishida, S., Terayama, K., Kojima, R., Takasu, K. & Okuno, Y. AI-Driven Synthetic Route Design Incorporated with Retrosynthesis Knowledge. *J. Chem. Inf. Model.* **62**, 1357–1367 (2022).
